# From fear to resilience: adolescents’ experiences of violence in inner-city Johannesburg, South Africa

**DOI:** 10.1186/s12889-017-4349-x

**Published:** 2017-07-04

**Authors:** Fiona Scorgie, Deborah Baron, Jonathan Stadler, Emilie Venables, Heena Brahmbhatt, Kristin Mmari, Sinead Delany-Moretlwe

**Affiliations:** 1 Wits Reproductive Health and HIV Institute (WRHI), Faculty of Health Sciences, University of the Witwatersrand, Johannesburg, South Africa; 2 Division of Social and Behavioural Sciences, School of Public Health and Family Medicine, University of Cape Town, Cape Town, South Africa; 3 Department of Population, Family and Reproductive Health, Bloomberg School of Public Health, Johns Hopkins University, Baltimore, MD USA

**Keywords:** Adolescents, Violence, Inner-city, Gender, Low-income, SOUTH Africa, Johannesburg

## Abstract

**Background:**

For adolescents growing up in poor urban South African settings, violence is often a part of daily life and has lasting effects on physical and mental health outcomes in adulthood. We conducted a qualitative study to document and understand the forms of interpersonal violence experienced by adolescents living in Hillbrow, Johannesburg. In this article, we explore how violence is experienced differently by adolescent boys and girls, how they conceptualise ‘dangerous’ and ‘safe’ spaces in their neighbourhood and what gaps exist in available services for youth in Hillbrow.

**Methods:**

The article draws on data collected in the formative phase of the ‘Wellbeing of Adolescents in Vulnerable Environments’ (WAVE) Study of challenges faced by adolescents (15–19 years) growing up in impoverished parts of five cities. This article reports on analysis using only data from the Johannesburg site. Using both purposive and snowball sampling to select participants, we conducted in-depth interviews (*n* = 20) and community mapping exercises with female (*n* = 19) and male (*n* = 20) adolescents living in Hillbrow, as well as key informant interviews with representatives of residential shelters, CBOs, and NGOs working with youth (*n* = 17). Transcripts were coded manually and analysed using an inductive thematic analysis approach.

**Results:**

Both girls and boys reported high exposure to witnessing violence and crime. For girls, the threat of sexual harassment and violence was pervasive, while boys feared local gangs, the threat of physical violence, and being drawn into substance-abuse. Home was largely a safe haven for boys, whereas for girls it was often a space of sexual violence, abuse and neglect. Some adolescents developed coping mechanisms, such as actively seeking out community theatres, churches and other places of sanctuary from violence. Community-based services and shelters that support adolescents reported a lack of resources, overall instability and difficulties networking effectively.

**Conclusions:**

Adolescents in Hillbrow commonly witnessed and had direct experience of many forms of violence in their environment, and these experiences differed markedly by gender. Interventions that build young peoples’ social capital and resilience are essential for reducing violence-related trauma and long-term health and social consequences for adolescents in this community.

## Background

South Africa is one of the most violent countries in the world: in 2005, as many as 39% of all deaths recorded at national mortuaries were due to violence [1] and an estimated 1.75 million people in the country seek healthcare annually for non-fatal injuries resulting from violence [2]. Death rates per 100,000 due to homicide and violence are five times as high as the global average (72.5 in South Africa, versus 14.0 globally in 2000) [3]. For young people growing up in poor communities, violence is often a part of daily life [4]. In a national household survey of over 3500 children aged 10–17 years, more than half (56.3%) reported lifetime physical abuse and 9% lifetime sexual abuse [5]. Data from the recent five-country WAVE study (Well-being of Adolescents in Vulnerable Environments) found that adolescents in Hillbrow, Johannesburg, were deeply concerned about their personal safety [6] – and for good reason. Two-thirds (67%) of adolescent males and 48% of adolescent females in this setting reported being a victim of violence in the past 12 months [7]. Prevalence of intimate-partner violence (IPV) in the past year was a staggering 36.6% among adolescent girls, the highest of all five cities in the study [8].

There are multiple causal pathways to violence in South Africa, with historical roots in “structural inequality, socio-cultural tolerance of violence, militarised masculinity, disrupted community and family life, and the erosion of social capital” [9]. Gender power inequity [10] and a history of racial discrimination and marginalisation also play an integral role. Furthermore, the poor are generally more vulnerable to violent crime than those in middle and upper income neighbourhoods owing to environmental factors such as poor public policing, poor street lighting, and overcrowding [1, 11].

Adolescents exposed to interpersonal violence^1^ – whether directly or indirectly, as witnesses – have substantial long-term health consequences, many of which remain unrecorded and unquantified [12]. Exposure also puts adolescents at higher risk of becoming either victims or perpetrators of further violence in adult life [13–16]. Experiencing violent crime is a major cause of post-traumatic stress syndrome (PTSS) and depression, and even living in high crime areas may induce fear and anxiety, negatively impacting on mental health in the long term [1]. A community study in Khayelitsha, Cape Town, found that 95% of children between the ages of 6 and 16 years had witnessed at least one violent event, and 22% met the criteria for PTSS, a substantially higher rate than that recorded among similar age cohorts in North America and Europe [17]. In inner-city Johannesburg, girls’ exposure to intimate-partner violence and non-partner sexual violence was associated with substance abuse, condom non-use, multiple sexual partners, pregnancy, transactional sex and poor mental health – with depression three times more likely in girls with experience of IPV than those without [8].

In the literature on violence, child abuse and social protection, more attention is being paid to the development of ‘resilience’ among victims [18–21]. In this context, resilience may be defined as the ability to develop “patterns of positive adaptation in the context of significant risk or adversity” [22]. While still nascent, the literature recognises that resilience is not only an individual personality trait but also a context-dependent interplay between vulnerability and protective factors in the social environment, such as the availability of social capital and cohesion [23, 24]. In theory, adolescents with greater resilience are at lower risk of developing PTSS, since they effectively have a ‘buffer’ to lessen the impact of violence [25], although it remains unclear how resilience is affected by instances of repeated exposure to violence. Also poorly understood is the question of how gender and other demographic factors shape levels of resilience among adolescents living in violent environments.

Much of the research on young people and violence in South Africa has come from low-income urban communities in the Cape [4, 25, 26], an area that has very specific socio-cultural dynamics relating to gang violence and poverty. In this article, we report on qualitative findings from the Johannesburg site of the WAVE study, a cross-sectional study of adolescents in low-income urban settings, conducted between 2011 and 2013. Specifically, we examine how violence – both in public spaces and in the home – is experienced differently by adolescent boys and girls living in Hillbrow, a high-density inner-city area. Placing adolescents’ own narratives at the centre of our analysis, we explore how they conceptualise ‘dangerous’ and ‘safe’ spaces in their neighbourhood, and how gender and space shape their everyday experiences of living here. By considering how adolescents make sense of and cope with living in a violent environment, we begin to unpack the forms that resilience might take in this setting. To our knowledge, this concept has not been used before to understand the lives of adolescents in South African inner-cities. Finally, we take a brief look at the social services and support available to vulnerable adolescents in Hillbrow, focusing on the gaps identified both by adolescents and by service providers.

## Methods

Data presented in this article were collected during formative research in the WAVE study, the full methods of which are described in detail elsewhere [6]. Study protocols, researcher training and consent procedures were standardised across sites. Methods included key informant interviews, in-depth interviews, community mapping and linked focus group discussions. In the Johannesburg site, community mapping involved only adolescents (15–19 years) living in Hillbrow at the time of the study (*n* = 39), as did the in-depth interviews (*n* = 20). Key informants (*n* = 17) included representatives of residential shelters, community-based organisations (CBOs), and non-governmental organisations (NGOs) working with youth.^2^

### Study setting

Hillbrow is a densely populated inner-city neighbourhood in central Johannesburg (an estimated 100,000 inhabitants in one square kilometre), with many high-rise apartment blocks, entertainment venues, small retail shops and informal traders. It is often the first place in which newcomers to the city settle. Hillbrow’s large migrant population incorporates economic migrants from other provinces of the country, as well as refugees and immigrants from across the continent and beyond. These newcomers are at the receiving end of pervasive regional xenophobia, and they are socially and economically marginalised, living in makeshift, overcrowded and often unsafe dwellings [27].

In quantitative findings of the WAVE study, many Johannesburg adolescents interviewed were living in unstable housing, often in single-parent households or lodging with relatives [28]. They reported low levels of support from caring family members and from neighbourhood resources, and had a much poorer perception of their physical environment than their counterparts in other cities [7]. Hillbrow, as they described it, was characterised by dirty and decayed buildings, overcrowded living conditions, garbage and sewage spilling onto the streets, and chronically neglected public infrastructure.

### Recruitment and data collection

During provisional mapping of shelters and services for young people in Hillbrow, the research team met with representatives of a diverse range of organisations working with youth in Johannesburg spanning the health, education, social welfare services and housing sectors. Through a combination of purposive and snowball sampling, these representatives were invited to participate as key informants, and were asked to introduce the researchers to adolescents who could potentially be invited to take part in the study. Additional adolescent participants were recruited directly from schools and youth shelters in the area by two trained female researchers, following the distribution of recruitment fliers in local streets, parks, churches, and the research clinic.

#### Community Mapping

Only adolescent boys (*n* = 20) and girls (*n* = 19) took part in these mapping activities. Eight mapping groups of between three and six participants were formed, and these met separately at the research clinic in Hillbrow or at a local residential shelter. The groups were gender distinct and divided by age (15–16 years old and 17–19 years old). Mapping was facilitated by two female researchers, trained by the lead qualitative investigator on the main WAVE study (KM), through the use of video clips and role-play exercises using the research instruments [6]. Discussion in the groups took place in English and two local languages – isiZulu and seSotho – and each session lasted between one and two hours. Participants were provided with flip chart paper, pens and stickers to produce the maps. Following the community mapping exercise, a focus group discussion was held with each group to collect additional information.

**Fig. 1:**
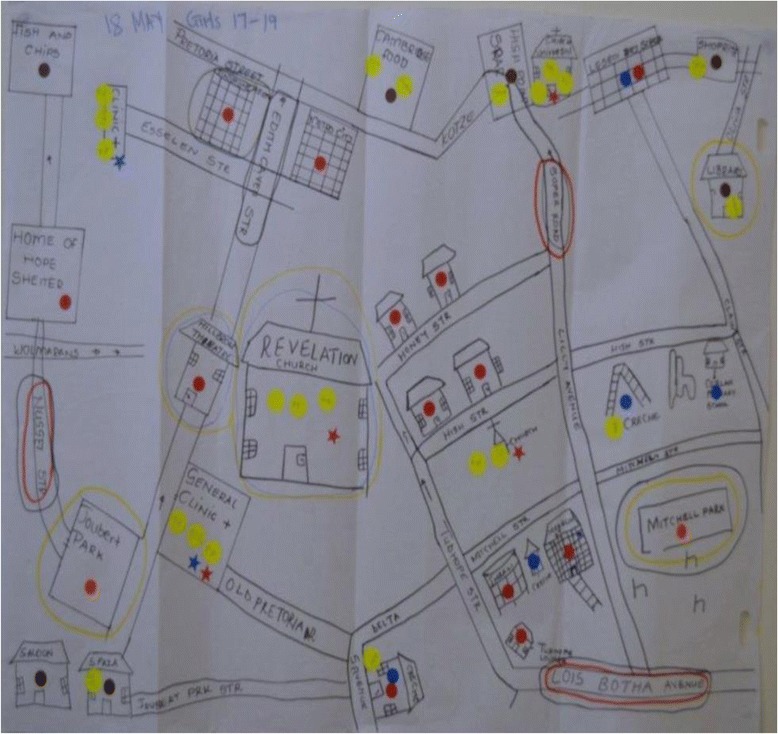
Girls’ map of Hillbrow. Legend: *Red* stickers show residential places; *blue* stickers = schools; *yellow* stickers = churches and religious buildings; *orange* stickers = places where young people hang out; *brown* stickers = libraries, cyber cafes. Circled in *yellow* = feel safe during day; circled in *blue* = feel safe at night; circled in *red* = don’t feel safe during day; circled in *black* = don’t feel safe at night

**Fig. 2:**
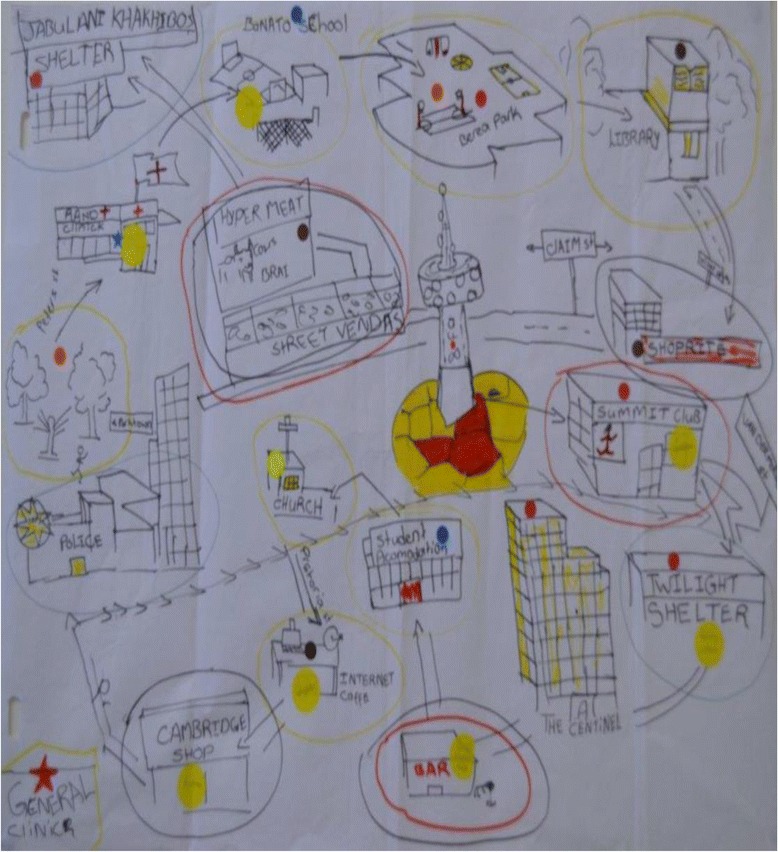
Boys’ map of Hillbrow. Legend: *Red* stickers show residential places; *blue* stickers = schools; *yellow* stickers = churches and religious buildings; *orange* stickers = places where young people hang out; *brown* stickers = libraries, cyber cafes. Circled in *yellow* = feel safe during day; circled in *blue* = feel safe at night; circled in *red* = don’t feel safe during day; circled in *black* = don’t feel safe at night

Participants were not expected to produce cartographically accurate maps of Hillbrow, but rather to draw a schematic representation of the neighbourhood based on how they experienced it as residents (See also [29, 30]). Recreational areas, apartment blocks, schools, libraries, and other venues were drawn, and participants circled those where they felt safe or unsafe by day and night. The activity created much animated discussion, which was audio-recorded. Images of two maps are included here as examples of the end products generated through this process (see Figs. 1 and 2).

#### In-depth interviews

In-depth interviews (IDI) were conducted with 20 adolescents at a research clinic in Hillbrow, using a semi-structured discussion guide with questions about personal perceptions and experiences of danger and safety in Hillbrow. In addition, key informants were interviewed about their experiences working with youth and about the experiences and life challenges faced by adolescents in the area. Interviews were facilitated by two trained female researchers from WRHI and lasted roughly an hour each. IDIs were conducted in English, and two local languages (IsiZulu and Sesotho). Notes were taken and interviews recorded using digital audio recorders.

### Data analysis

Audio files of the IDIs and community mapping discussions were transcribed and translated into English, where necessary, with quality checks on the translations conducted by the site Principal Investigator. Analysis was carried out manually by two authors (FS and DB), who read the transcripts to identify initial themes and develop a coding structure using an inductive thematic analysis approach [31]. Transcripts of IDIs with adolescents and community mapping activities were combined for the purposes of analysis and then coded using a set of key themes, with additional themes added iteratively as the analysis proceeded. Girls’ transcripts were analysed separately from boys’ transcripts, and the coding structure was set up to enable comparison of adolescents of different ages. Coding of transcripts concluded when all data had been assigned to a code and saturation achieved.

The final set of key themes included: public violence and crime; gang violence; sexual abuse; nature of home life; greatest concerns or worries; positive/negative aspects of living in Hillbrow; reflections on how girls’ and boys’ experiences of violence differed; availability of local support services; and safe spaces. Analysis of the maps developed during community mapping exercises involved assessment of their layout, a count of ‘safe’ and ‘dangerous’ spaces, and their physical distribution across the terrain of Hillbrow. Key coding themes from analysis of key informant interview transcripts included: nature and scope of work; interaction with adolescents; greatest challenges in service provision; and the impact of the Hillbrow environment on young residents.

Our analysis was also partly informed by narrative analysis, taking the position that adolescents’ accounts about everyday life are not solely descriptive but are meaningful acts, potentially both socially powerful and personally empowering [32, 33]. As such, their narratives are commentaries about the lived world as well as idealised worlds [34], and provide insight into young peoples’ coping strategies, resistance to victimisation, and aspirations for the future.

## Results

Four distinct thematic areas emerged from an analysis of our findings. Following a brief overview of sample characteristics, we tackle each of these themes below. Firstly, we report on the multiple forms of violence experienced by young people in Hillbrow’s public spaces, followed by a look at what domestic life holds for them: is home necessarily a safe haven in this setting? Then, we consider how Hillbrow’s adolescents identify ‘safe’ and ‘unsafe’ areas in the neighbourhood, and navigate these spaces to minimise risk. This section reflects on how these young people make sense of their environment and deal with negative stereotypes about Hillbrow in surprisingly positive ways. Finally, we present data on the availability and quality of support services in Hillbrow, from the perspectives of service providers and of the young people they are intended to serve.

### Sample overview

In total, 59 adolescents took part in the study (community mapping = 39; IDIs = 20). Equal numbers of girls and boys took part in the IDIs, while the community mapping involved 19 girls and 20 boys. On average, the 17 key informants interviewed (10 female, 7 male) had worked with youth for 10 years, with experience ranging from 1 to 36 years.

Among the adolescent IDI participants, four boys and three girls reported having at least one deceased parent. Some participants lived with relatives as their parents either could not afford to take care of them or lived too far away, and two of the girls lived in a shelter because they had been expelled from home by relatives. Virtually all participants made some reference to financial hardship in their households, but for at least half of the girls interviewed, these hardships were acute: where money for food, school fees and rent was often inadequate. Two girls had recently experienced interrupted schooling owing to a lack of finances. Earning a small income through independent, part-time work appeared to be more common among boys (fixing cars, selling flowers or recording music for local DJs) than girls (braiding hair). Two girls mentioned receipt of state child support grants, but claimed these funds were being collected by relatives and used for other purposes.

### “Outside these walls it’s dangerous”: violence in Hillbrow’s public spaces

In the narratives of adolescent participants, the social environment of Hillbrow was dominated by violent crime, substance abuse, and sex workers, a place where drugs were routinely sold in the open. Police intervention in Hillbrow was described by most as virtually non-existent, with the only effective protection against violent crime being that offered by armed guards hired by private security companies and reserved for the neighbourhood’s entertainment venues. Drug- and alcohol-fuelled fighting in public spaces was reportedly a common occurrence, along with mugging and petty theft. One boy recounted his experience of the latter just a few days prior to the interview.

*“…they took my cell phone from me and they had a knife and that happened when I was coming back from rugby practice. I was coming there by Shoprite and it was dark and I think it was probably around eight thirty in the evening…I was traumatised…I was alone and I was shocked.”* (Tshepo,^3^ 15 year-old boy)

Participation in petty crime and substance abuse was allegedly required for membership to some male gangs that populated the Hillbrow landscape, intimidating people, stealing cell-phones and instigating violent clashes. A gang known as the ‘Vandals’ apparently used plastic rulers to search for phones hidden on the bodies of their victims. Their *modus operandi* was simple and effective: *“They take people’s phones, they hit people, they ask you for money, you say no, the next thing they hit you”* (Karabo, 16 year-old girl). Another gang known as the ‘CMFs’ (“Crazy Mother Fuckers”) were known for stealing and stabbing. They were “*…always drinking alcohol and if you quarrel with one of them they all come to you because their slogan is ‘touch one, touch all’”* (Busi, 16 year-old girl). Participants described how the determination of certain gangs to protect their turf meant that spatially, Hillbrow was divided into a number of tightly controlled ‘no-go’ areas. Those who strayed into their territory – whether members of other gangs or not – were likely to be punished with threatened or actual physical violence.

Not all gangs were regarded as violent, however. Local groups such as the ‘Pantsulas’, ‘Bourgeois’ and ‘Hip-hoppers’ were apparently better known for their music and clothing preferences, and for characteristic styles of dancing. But intense competition among local gangs, even those defined more by dancing than by a penchant for violence, meant that physical conflict routinely erupted between them, particularly when sexual liaisons developed across gang boundaries. Boys explained that young people who were simply bystanders could inadvertently be drawn into these competitive and intrinsically violent interactions. One popular gang in the neighbourhood, they claimed, was the ‘*Izikhothane’,* modelled on a notorious youth sub-culture that originated in Soweto in the early 2000s [35]. *Izikhothane* members would wear luxury branded clothes and nihilistically flaunt wealth they did not have. They were said to tear up banknotes and clothes “*and throw [them] at you”.* Boys said they felt “*ashamed*” when confronted with *Izikhothane*, and hinted at how the gang ethos fused local constructions of masculinity with the glorification of ostentatious displays of consumerism:

*“…some of us want to live a peaceful life and it’s not a nice thing if you go out there and someone says ‘look at this one, the poor one, he’s got nothing and we’ve got this and that’. And that won’t make you feel good about yourself…you will end up beating them up because that person will be embarrassing you. And it would be worse if there were girls around because you don’t want to be embarrassed in front of women.”* (Boys CM 2402)

While none of the boys reported membership to a gang or direct experience of violence inflicted by gang members, some had witnessed others being attacked and mugged. They expressed anxiety about the need to avoid encountering these gangs in their day-to-day interactions. Overall, boys’ detailed descriptions of adult male gang behaviour revealed an acute awareness of how their presence in Hillbrow was impacting on the ability of all residents to feel safe.

By contrast, girls’ narratives focused much more on their personal experiences of sexual harassment in the public spaces of Hillbrow – mentioned by almost all girls in the IDIs. This was commonly referred to as being “pulled on the street”, and reflected how the simple act of moving around in public was a deeply gendered one. Girls in a community mapping group explained,

*“When you walk around they [men] call you…Yes and say things such as ‘my size, hey my size come here’, I will give you money.”* (Girls CM 0304)

Others spoke of their reluctance to visit bars in the area, because they could expect to encounter men who were “*too touchy-touchy [laughter]”* (Girls CM 0404). Rose (17 year-old girl) described being targeted sexually by the leader of a local gang:

*“Every time I come to Hillbrow, I am scared that I might meet him. […] When I see him I have to kiss him, like really…I’m scared he will hit me, like really bad. […] Yes! ‘I will slap you, if you don’t kiss me now like really I will point you, I will take out my knife, I will stab you…’.”*

Many adolescents drew connections between sexual violence, transactional sex, and the drug trade – which they believed had been boosted in the area by the presence of foreign men. Indeed, allegations that foreigners were the primary source of crime in Hillbrow were common. Portia (19 year-old girl) spoke about harassment by “*foreign guys selling drugs*” in public:

*“…like if you are walking by them they will be pulling you, [saying] ‘come here my friend I will give you this, anything you want in this world.’ What is that? ‘Cos the guy knows he sells drugs, all he wants to do is spoil my life. You know, he wants to sleep with me…”*

This perception contrasted starkly with key informants’ main concern in relation to foreigners in Hillbrow, namely, that xenophobic attitudes were preventing these migrants from accessing local health services.

Finally, in addition to direct, personal experiences of violence, both boys and girls reported commonly witnessing acts of violence in public.

*“…we see a lot of things. We see people fighting, drinking, we see pornography live. We just see it there, people raping each other …”* (Cécile, 16 year-old girl)

While boys residing at the Twilight Shelter returned by 18 h00 every evening – for their own safety – they still frequently witnessed violence from the confines of the shelter; they *“would see people fighting outside, if looking out the window*” (Boys CM 0302). Unsurprisingly, the witnessing of such violence was said to have clear mental health consequences:

*“Kids experience a lot of fighting in the community, when someone is fighting someone, and taking the bottle to strike someone with this bottle, that is not something good for a child to see. It goes deep into them and it depresses them.”* (KI09; male orphanage director)

### “They know that if they speak something, uncle will kill me”: Violence in the home

Compared to descriptions of violence in the *public* spaces of Hillbrow, violence in the home was characterised by an even more profound gender asymmetry, in that it was raised almost exclusively by girls. Their stories took three broad forms: direct experiences of sexual violence; witnessing domestic violence; and experiencing emotional abuse and neglect.

Accounts of sexual violence in the home mostly involved perpetrators who were known to the girls. Fifteen year old Thembi, who was perinatally infected with HIV and currently not attending school, stayed with her aunt and uncle following her mother’s death from AIDS. While her aunt was away from home for some time, Thembi was subjected to repeated rape by her uncle –

*“…one evening [he] came home and started touching my private parts and told me that he is going to teach me how to please a man and he raped me. And this happened several times until I told my aunt about it and she didn’t believe me, but took me to the clinic …she asked that I be given family planning injection.”* (Thembi, 15 year-old girl)

Efforts to tell other adults in her surroundings, including a neighbour, led to further victimisation:

*“ I told her what my uncle is doing to me and she asked my aunt about it and she [the aunt] said that I am lying, that I want to take her man from her, so she beat me after the lady left… she told me that I am lying and called me a ‘whore’.”* (Thembi)

Thembi’s was the only direct experience of rape reported by participants, but it nonetheless highlights a more general absence of positive adult role models for young people in this area, including recourse for young people experiencing sexual violence in their homes. It is perhaps not surprising that key informants remarked on the under-reporting of abuse in the home as a significant challenge in their work. The director of a local orphanage alluded to even greater violence that awaited victims who spoke out about their abuse, although did not provide specific examples of such extreme retribution:

*“You see the problem here is that so many girls have these problems [sexual abuse] and they don’t talk. They keep it a secret…these children are small, but they are scared of people. They know that if they speak something, uncle will kill me…The children are living in fear.”* (KI09, male orphanage director)

A second form of violence in the home involved the witnessing of domestic violence. Rose described watching her sister being beaten up by her partner, to the point where she was unable to walk. Although the police were called, “*they didn’t come*”, forcing Rose to go to the police station the following morning to open a case of assault. Similarly, Busi described her failed attempts to intervene when her father was abusive:

*“My dad abuses my mom when he is drunk… He swears at my mom and it happens every time he is drunk...It makes me feel terrible, bad and sad. I try to stop him and he always tells me not to defend my mother because he will hit me.”* (Busi, 16 year-old girl)

Nineteen year old Sello recalled witnessing his father’s violent attacks on his mother over the course of several years, eventually culminating in his parents’ separation. Sello was the only boy who reported domestic violence in his home.

Thirdly, and perhaps less easy to categorise, was a form of emotional abuse and neglect experienced at home by girls such as Lerato, Cécile and Zama, who had been separated from their parents, either by death, poverty or conflict in the family. As a consequence, they stayed with relatives in somewhat precarious home circumstances, were often made to carry out the majority of the housework, including caring for younger siblings, and at times made to feel unwanted. Living in a shelter at the time of the interview, Cécile had been thrown out of her home by her step-father who suspected her of using witchcraft against his own children. She then went to stay with an aunt,

*“I had to clean the house, wash her clothes, her son’s clothes, her daughter’s clothes and her husband’s clothes and I had to cook and everything, and sometimes she would not give me food.”* (Cécile, 16 year-old girl)

Cécile then left her aunt’s house and moved into the shelter. In the interview, she reflected that it *“hurts a lot”* to not live with her mother and siblings anymore, and that growing up without parental guidance was a real challenge.

*“…living life away from them, it’s painful because life in this place is hard. You get into the adolescent stage and you don’t know who to trust, who to go to give you advice then the things people do here, you don’t know if this is good or this is bad.”* (Cécile)

This absence of a positive adult role model was also keenly felt by 17 year-old Zama, who lived with her sister and her sister’s partner, whose emotional abuse led her to frequent thoughts about running away. Her mother died in 2000, and she has never met her father.

*“At times I get angry when I am at home because we live with my sister’s boyfriend and I don’t like him much, we don’t get along...When he gets home drunk he will start telling my sister about how useless I am and that he doesn’t like me.”* (Zama, 17 year-old girl)

None of the boys reported having being rejected or chased away by their families, nor did they report experiencing physical abuse in the home. In the final sections below, we explore how adolescents in our study have responded to these experiences of violence – whether remaining silent, tapping into support services in their environment, or forging their own strategies for coping and staying safe. Such responses are crafted against the backdrop of how residents of Hillbrow imagine and interact with its physical spaces.

### Geographies of danger: finding safe spaces and reframing violent realities

At first glance, the community maps produced by adolescent participants in our study simply lay out the spatial arrangement of Hillbrow: the streets, apartment buildings, shops, the police station, churches, parks, and schools, and so on. Closer examination reveals that they also depict a ‘geography of personal danger and safety’. As the social realities of living in Hillbrow were plotted onto representations of its physical spaces, isolated pockets of safety emerged amidst this complex terrain. As with the exposure to violence in public and at home, there were clear gendered differences in how this precarious geography was lived and imagined.

Overall, boys’ and girls’ maps differed mainly in relation to the number and dispersal of ‘safe’ spaces identified across Hillbrow. Figures 1 and 2 illustrate this pattern well: the girls’ map (Fig. 1) identifies only a few places concentrated in a small area of the neighbourhood as ‘safe’, suggesting that girls’ movement beyond this cluster is probably quite limited. Areas identified as ‘unsafe’ by the girls are specific sections of streets that are main thoroughfares or access/exit points to Hillbrow, which in turn corresponds to their complaints of being “pulled on the street” by men, and of needing to find alternative routes when “being sent” to run errands, or when walking to and from school (see below). The boys’ map in Fig. 2, by contrast, identifies more locations as ‘safe’, and these are scattered across a wider area. Retail and entertainment venues, which pose the risk of muggings and alcohol-fuelled violence, were cited most as ‘unsafe’. Age did not appear to be a strong factor shaping these maps – except in one respect, namely, that older girls (17–19) seemed more aware than their younger counterparts of which entertainment venues served alcohol – described as dangerous but also enticing – and hinted at their own patronage of these venues.

Since virtually all public spaces were regarded as dangerous after dark, few participants had direct experience of the city at night, particularly those who lived in shelters, which had strict curfews. For girls, freedom of movement was also limited after school and on weekends to their own homes, the homes of friends, churches, or well-populated shopping areas. The observation of sixteen-year old Busi was a common one: *“I am only with my friends on weekends and we never go anywhere. We just play around our flat”*. Even one of the boys commented on the apparent ‘informal curfew’ that applied to girls in Hillbrow and limited their mobility:

*“Girls have to be home around six because of their safety, while we as boys can be home around ten in the evening.”* (Joe, 16 year-old boy)

The Hillbrow Theatre was a space identified by all girls’ mapping groups as ‘safe’. This complex, which hosts after-school drama, dance and music programmes for young people in the inner-city, is a popular spot, particularly in the afternoons and on weekends. For boys, libraries and internet cafes were popular retreats, along with parks, which enabled them to *“relax”* and *“refresh your mind”.*

*“…because during the day there are people there [in Berea Park] doing different activities such as exercising and all that…the atmosphere is great…it makes you be at ease. And there are security guards there.”* (Boys CM 2402b)

Boys tended to avoid places where they were likely to encounter peer pressure to engage in physical conflict or substance use. One boy even framed this strategy as a need to be safe not only from “*the outside world*” but also “*from my own danger*” (Boys CM 2402). Churches were identified as potential ‘sanctuaries’ regardless of gender, while schools were only mentioned by boys as offering protection from outside dangers. Girls cited instances of sexual harassment by schoolmates, suggesting that in this respect at least, schools were no different to other public spaces, where such harassment was the norm.

While one might expect young people to regard their homes as ‘safe spaces’, importantly, this association was heavily contested within the girls’ mapping groups. Those who asserted that they felt safe *“at home”* or *“in flats”* were almost always challenged by fellow group members who had experienced otherwise. One girl specified, *“I feel safe only on my floor, because we lock”* (Girls CM 1805), while others contended, *“no, I don’t feel safe at our flats”* (Girls CM 0404) and even claimed, *“your flat is full of thugs…they mug you around there*” (Girls CM 0404). By contrast, boys from a shelter were unanimous in identifying this home as a safe haven from street violence, “*from people who might mug you and also from gunshots”.* Another said,

*“[In Twilight Shelter] there is always protection… We are safe from drugs, crossfires, crime, human trafficking and a lot of bad things out there.”* (Boys CM 2402)

Encounters with potentially violent petty criminals on the streets of Hillbrow were seen as almost inevitable, but steering clear of well-known dangerous routes during the day was a common tactic, particularly for girls: 15 year-old Lerato’s mother could not allay her fears of “*kids who stay at Umshangani Park*”, whom she passed when running errands for adults in her household, “*because she [her mother] knows that if they come for me then they’ll come.”* Lerato’s only strategy – and one that demonstrated agency – was to “*take a different direction*” on her route in the hope of avoiding them.

For others, coping with life in a violent environment was easier when one had become desensitised to its effects. Vusi (15 year-old boy) had witnessed so much violence in his neighbourhood it had become ‘normal’:

*“I guess for me it’s normal because I have lived here most of my life. Like some of my friends when they come here they tell me that Hillbrow is dangerous and stuff like that, but for me I guess I know that it can be dangerous. I have seen people die here and whatever but you know, it’s something I am used to.”*

Vusi’s apparent nonchalance was not shared by all. Some participants – girls in particular – alluded to high levels of anxiety and stress and indicated that coping with constant fear was a challenge. Sixteen year old Busi reflected that: *“There is nothing easy about staying in Hillbrow, everything is difficult”,* while Thembi’s struggles to cope with trauma following sexual violence were heightened by the intense isolation and lack of support she experienced living at a shelter. She occasionally visited an aunt and some cousins on weekends, but this was about to change:

*“…they said they don’t want me there anymore and they told the social worker that. […] No one visits me. I don’t have friends.”* (Thembi)

Despite this somewhat grim picture, however, other narratives embodied a strong sense of survival against the odds, and even an expression of community pride in Hillbrow. Tshepo (15 year-old boy) reckoned, *“it’s not safe living in Hillbrow but we survive”*, while Sandile (18 year-old boy) claimed, *“I enjoy it a lot. It’s a good place even though there is crime”.* In contrast to those who blamed foreign migrants for the unsafe streets of Hillbrow, other participants embraced the diversity of this neighbourhood, crediting its cosmopolitan make-up with broadening their exposure to a variety of people, languages and diverse points of view:

*“…there are lots of opportunities and it’s all about what you make out those opportunities… So Hillbrow is a fantastic place to us…But it’s all about what you make out of it.”* (Boys CM 2402b)

Mbali (18 year-old girl), who had moved from Kwa-Zulu Natal to live with an aunt in Hillbrow, stated that *“staying in places like these, where there are lots of people opens your mind; it’s not the same as living in the rural areas”.* In a girls’ mapping group, one participant even exclaimed, “*it’s a cool place to be….I am proudly Hillbrowian! (laughter)”* (Girls CM 0304).

Regardless of adolescents’ own thoughts on Hillbrow, however, they were often confronted with the negative perceptions held by others. As one girl put it, “*there is a perception that when you say you stay in Hillbrow, you prostitute yourself or you drink alcohol or you take drugs*” (Mbali). Most participants recounted being teased by peers who lived in townships, and having to either ignore the teasing, defend their reputations, or even pretend that they lived elsewhere – acts that in themselves became forms of agency and resilience. Vusi was one of the few who seemed able to brush off these remarks:

*“…at school people call me a drug dealer and say things like I am a criminal and stuff like that, you know, just the typical stereotype. […]Well I don’t take it as like insulting or something like that. It’s just I take it as a joke.”*

Evidence of self-motivation and agency in the face of adversity appeared also in the narrative of Cécile (16 years), a young Congolese woman living in a Hillbrow shelter for girls. A sense of hope was palpable in her vision for the future, which involved a commitment to improve both the shelter and the neighbourhood that had taken her in.

*“I want to be an attorney because of the things I see here. I see that there is lot of injustice and unfair trials in this place, so I want to make a difference and my first pay check should be given to this [shelter] to help it become better.”* (Cécile)

In the long run, these perspectives point to promising signs of resilience among some adolescents – both male and female – for it is the ability to reframe their surroundings in more positive ways that may ultimately serve to lessen the risk, fear and trauma associated with living in the inner-city.

### An imperfect stop-gap: support services for vulnerable youth

Beyond the construction of certain physical spaces as ‘safe’, however, adolescent participants also commented on the scarcity of external support services and interventions available to help them should they ever become victims of violence. A dominant theme here was that while psychosocial support – particularly counselling services – was known to exist in theory, many adolescents were either not aware of how and where these services could be accessed, or (based on prior experience) they doubted their ability to meet their needs. Some were unaware that services targeting youth existed in Hillbrow at all.

From the perspective of service providers, however, the nature of this gap between demand for and supply of psychosocial services was more complex than ‘mere’ lack of awareness. One key informant who had grown up in the neighbourhood was running a peer education organisation in Hillbrow which was struggling to secure the necessary funding and support. In his estimation, adult residents in Hillbrow did not “*have time to come and donate and see what their children are getting up to when they are out”.* For him, there was no shortage of potential resources – organisations, services and safe spaces – on which young people in Hillbrow could draw. But these resources were under-funded and under-used, owing to chronic apathy and a series of “disconnects” between the services and their target populations.

*“…there is just something that’s lacking between the service provider and the people needing this service. You know people still need to be convinced as to why do we need this service.”* (KI10; male community organisation leader)

Other key informants spoke of numerous challenges facing services that attempt to target local youth with recreational activities. These included chronic under-funding and inadequate coordination with the police and other social service agencies. Key informants working in psychosocial services noted the continuing stigma around sexual abuse, particularly for male victims, which accounted for the profound under-reporting to police services, and the low uptake of sexual abuse counselling. A female manager at a national counselling organisation commented on her observation of serious mental health problems among youth. Among the greatest problems was a pattern of suicidal ideation and self-harming behaviour, which she attributed to being “*exposed to things that they don’t have the emotional capacity to deal with”*. Often undiagnosed, even when these cases do come to light, “*there are no resources*” to support referrals into proper care. This was also true of referrals for young people with substance abuse problems. Without prompting, Sandile (18 year-old boy) expressed awareness that his own drinking had become harmful – “*I drink too much, but can’t seem to stop”* – and which he was attempting to address alone.

Echoing the key informant who was concerned about self-harming behaviour, another noted that young boys *“…don’t know what to do with their feelings…it leads to quite a lot of underlying anger simmering from hopelessness”* (KI016; female counsellor). While we did not explicitly pose the question of young people themselves perpetrating violence, examples of this did emerge in the data, particularly in relation to children living on the streets:

*“…when they first arrive, you will see that a child [is] not violent, but once they start staying on the streets they learn all those tricks to survive on the streets, maybe they are fighting to wash somebody’s car…they are also fighting to carry somebody’ bag. It's a serious issue. You will hardly find a child who has stayed on the streets who has never fought with any other child.”* (KI12; male shelter worker)

Other key informants considered existing counselling and support services in Hillbrow to be *“geared for adults”* and therefore not youth-friendly, leading to further limitations around access and impact. On a similar theme, Rose (aged 17) believed that what young people in Hillbrow needed most to help them cope with the challenges of their environment and build resilience, were opportunities for collective problem-solving under the guidance of adult mentors. She offered specific recommendations for these mentors to lead “*groups that would motivate youth as to how they should handle life*”. Her suggestion highlights one way in which existing services and organisations could become more responsive to the needs of adolescents: by addressing the longer term problem of absent positive adult role models, rather than only providing a temporary ‘stop-gap’ service for youth who are victims of violence.

## Discussion

Adolescents’ narratives about everyday life in Hillbrow painted a picture of pervasive violence, with armed muggings, gang-related violence, crime linked to substance use, sexual assault, and domestic violence all unfolding against the background of flawed public policing and social service systems, which have largely failed the people of Hillbrow. Local service providers describe valiant efforts to run programmes for youth while facing limited resources, virtually non-existent engagement from adults in the community, and at times, direct obstruction from public sector representatives. The broader context is one in which a weakened civil society is confronting a growing lack of infrastructure and accountability. Many NGOs and CBOs in South Africa experienced serious funding declines after the democratic transition in 1994. Rising antagonism from government, particularly intense in the last decade, has further undermined the ability of these organisations to effectively advocate on behalf of the increasingly marginalised communities they represent [36]. Such underlying structural factors compound the poverty-linked vulnerability of young people in Hillbrow, but could also be generalizable to other urban slums – particularly those in Latin America, which mirrors South Africa’s levels of poverty and inequality, and has similar examples of poorly controlled urbanisation and spatial segregation [37]. Indeed, evidence emerging from our study suggests that everyday violence in Hillbrow is “invisible” and “normalised” in much the same way that Scheper-Hughes has shown in her study of violence in impoverished, inner-city Brazil [38].

Overall, the home circumstances of the girls in this study appeared less stable and more burdensome than those of their male counterparts, with the effects of poverty, alcohol abuse, gender-based violence, domestic responsibilities and the absence of adult role models strikingly evident in their descriptions of everyday life. Indeed, the findings on orphanhood and the lack of a secure home for a sizeable portion of our sample warrant further investigation. We may ask whether some of the young people living in unstable housing and shelters in the inner-city are there because extended families are struggling to absorb those orphaned by the HIV epidemic. From the limited data presented here, we might also posit that in this setting, boys are more successful than girls at securing an independent income – however modest – whereas girls appear to be more dependent on others for financial support. This may be due in part to the boys’ greater freedom of mobility in Hillbrow’s public spaces. It also follows a pattern of traditional gender roles, in which men are expected to be ‘providers’ for women, and the ability to earn an income becomes a key marker of masculinity [39]. The stereotype emerges even in the ethos of some male gangs that appear to have infiltrated the social fabric of Hillbrow, which reward men who can show evidence of wealth, and shame those who cannot. Local gender norms appear to reward male aggression and female disempowerment, in turn fuelling male gangs and deepening girls’ vulnerability to sexual violence. The question then becomes: in settings of heightened vulnerability for young people, would compliance with these gendered roles potentially safeguard their access to networks and sources of social capital, thereby shoring up resilience – or render them even more vulnerable?

Studies of low-income urban communities in the Cape, where male gang activity and community vigilantism are rife, have found that boys experience higher levels of violence-related trauma than girls in the same areas [4]. Our findings on male gangs in Hillbrow suggest a need to revisit the assumption that gang-related violence is restricted to the Cape. And while we did not measure trauma levels among participants per se, the qualitative findings on the high burden of sexual violence experienced by girls suggest that Hillbrow may be a place where this gender asymmetry is inverted. In this respect, our findings echo the quantitative analysis in the larger WAVE study, which found that adolescents in the Johannesburg site displayed extremely high rates of depression (41.1% of males and 44.6% of females above the cut-off point for mild depressive symptomology) and PTSD symptoms (54.5% of males and 67.0% of females) [28]. The experiences of girls in our study highlight the extent to which their sexual availability for men is assumed is this setting, along with the expectation that girls will occupy the position of domestic servitude within the household reserved for them.

In the absence of positive adult role models, where families are disrupted and violence against women and children becomes normalised, the young people of Hillbrow are left in a precarious position. Yet girls’ lack of personal safety in the home and the experience of witnessing domestic violence point to generations of trauma experience. Repeated cycles of poverty and violence over time create environments that entrench negative social norms and expectations across generations – in spite of the aspirational legislation created after 1994 to counter this. The challenge remains one of generating structural changes that can break these harmful patterns and build new positive social norms. Arguably, this requires structural interventions that directly engage with the gendered patterns of violence in this setting and the gendered differences in local alcohol and drug use, as suggested by this study and confirmed by other research [40].

Outside of the home, individual strategies developed by both girls and boys in Hillbrow to minimise risks of violence are similar to those documented more than 15 years ago among women working in this area (mainly rubbish collectors and sex workers) [41]. To cope with the daily risk (and fear) of rape, they created “avoidance zones” around particular streets and public spaces, largely based on prior experience. Adolescents in our study similarly constructed ‘safe’ spaces amidst a plethora of areas in the neighbourhood known to be dangerous, and structured their daily movements accordingly, to minimise their risk of exposure to violence. These strategies have limited use in the long term, however, as they restrict the physical mobility of young people – particularly of girls, who are subjected to sexual harassment in public spaces in this setting in ways that boys simply do not experience. This limited mobility has implications for girls’ ability to access information, care and other public resources, constraining their access to social networks and other sources of support to the few areas of Hillbrow in which they feel ‘safe’.

How do we craft appropriate and effective responses to this situation? Aside from the critical need for improvements in the physical environment, properly resourced, gender sensitive and youth-appropriate services are also required to assist young people who have been exposed to violence. Arguably, this should take the form of interventions to build social capital and deepen adolescents’ existing capacities for resilience, rather than being content with efforts to ameliorate its effects at an individual level alone, such as trauma counselling [42, 43]. The success of such resilience-based interventions will depend largely on the availability of sustained adult and community support. This could take the form of structured adult mentorship programmes, which may help to protect against trauma and risk-taking behaviours later in life: research has shown that a relationship with a caring and committed adult “can buffer a young person who grows up in a violent and abusive environment from its consequences” [44].

In reflecting upon adolescents’ narratives about Hillbrow, we were struck by their descriptions of a community that yields diverse opportunities for young people, and even elicits expressions of neighbourhood pride. These sentiments existed side-by-side with stories of streets marked by violence and fear. The re-framing of Hillbrow as a “cool place to be”, as a culturally-diverse neighbourhood that was a “good place even though there is crime”, hints at adolescents’ capacity for resilience and strength in an environment that would appear to offer little more than hopelessness and despair. These indications of “everyday resilience” [45] perhaps offer some challenge to conventional understandings of post-traumatic stress syndrome as an inevitable response to violence, a response that “underestimates the human capacity not only to survive, but to thrive, during and following states of emergency, extreme adversity, and everyday as well as extraordinary violence” [45].

### Study limitations

A number of limitations must be borne in mind when considering our findings. Firstly, the sample of adolescents interviewed for the study was small and not randomly selected, thus limiting the extent to which broader generalisations can be made. Secondly, we did not ask these participants directly how they coped with living in a violent environment, and had to rely largely on implicit references to coping in order to understand the forms of resilience emerging in this setting. Finally, our assessment of local services for adolescent victims of violence draws entirely on the perspectives of adolescent participants and a small sample of key informants working in Hillbrow; analysis of these data would undeniably have benefited from a more comprehensive audit and formal evaluation of such services.

## Conclusions

Our study reaffirms the agency of adolescents in Hillbrow, even in the contexts of vulnerability currently at play in these inner-city areas, where exposure to violence is part of daily life. Young people are reaching out to find positive recreational spaces, and searching for sanctuaries from violence in their neighbourhoods to provide them with a sense of belonging and collective affirmation. Nascent signs of underlying resilience among adolescents could be leveraged and expanded through appropriate and adequately resourced community-level interventions, located within safe spaces close to where they live. For these interventions to succeed, strong commitment from local and national stakeholders are needed, along with positive adult role models and regenerated public institutions, fully accountable to the communities they serve.

## Abbreviations

CBO: Community-based organisation
CM: Community mapping
IDI: In-depth interview
IPV: Intimate partner violence
KI: Key informant
NGO: Non-governmental organisation
PTSD/S: Post-traumatic stress disorder/syndrome
WAVE: Well-being of adolescents in vulnerable environments study

## Footnotes

1 In this article, we use the WHO definition of interpersonal violence: “the intentional use of physical force or power, threatened or actual, against another person or against a group or community that results in or has a high likelihood of resulting in injury, death, psychological harm, maldevelopment, or deprivation” (Dahlberg LL, Krug EG. Violence: a global public health problem. In: Krug EG, Dahlberg LL, Mercy JA, Zwi AB, Lozano R, editors. *World report on violence and health*. Geneva (Switzerland): World Health Organization; 2002. p. 1–21).

2 A Photovoice exercise with 10 adolescents was also carried out as part of this phase of the study. We do not present any results from this exercise here.

3 All participant names in this article are pseudonyms. “Boys CM” and “Girls CM” refers to Boys’ and Girls’ Community Mapping groups respectively. “KI” refers to Key Informant.
